# The Human Gut and Dietary Salt: The *Bacteroides*/*Prevotella* Ratio as a Potential Marker of Sodium Intake and Beyond

**DOI:** 10.3390/nu16070942

**Published:** 2024-03-25

**Authors:** Omololu Fagunwa, Kirsty Davies, Jane Bradbury

**Affiliations:** 1 Institute for Global Food Security, School of Biological Sciences, Queen’s University Belfast, Belfast BT9 5DL, UK; 2 School of Applied Sciences, University of Huddersfield, Huddersfield HD1 3DH, UK; kirsty.davies@hud.ac.uk; 3 School of Medicine, Edge Hill University, Ormskirk L39 4QP, UK; jane.bradbury@edgehill.ac.uk

**Keywords:** microbiome, gut, microbial ratios, ENaC, TspO, sodium intake, cholesterol, *Bacteroides*, *Prevotella*

## Abstract

The gut microbiota is a dynamic ecosystem that plays a pivotal role in maintaining host health. The perturbation of these microbes has been linked to several health conditions. Hence, they have emerged as promising targets for understanding and promoting good health. Despite the growing body of research on the role of sodium in health, its effects on the human gut microbiome remain under-explored. Here, using nutrition and metagenomics methods, we investigate the influence of dietary sodium intake and alterations of the human gut microbiota. We found that a high-sodium diet (HSD) altered the gut microbiota composition with a significant reduction in *Bacteroides* and inverse increase in *Prevotella* compared to a low-sodium diet (LSD). However, there is no clear distinction in the Firmicutes/Bacteroidetes (F/B) ratio between the two diet types. Metabolic pathway reconstruction revealed the presence of sodium reabsorption genes in the HSD, but not LSD. Since it is currently difficult in microbiome studies to confidently associate the F/B ratio with what is considered healthy (e.g., low sodium) or unhealthy (e.g., high sodium), we suggest that the use of a genus-based ratio such as the *Bacteroides*/*Prevotella* (B/P) ratio may be more beneficial for the application of microbiome studies in health.

## 1. Introduction

The gut microbiota is an intricate and dynamic ecosystem, hosting an array of microorganisms that play a pivotal role in maintaining host health [1,2]. The perturbation of these microbes is linked to several conditions, such as cardiovascular diseases [3,4], respiratory diseases [5,6,7], neurological diseases [8,9], gastrointestinal diseases [10,11], endocrine diseases [12,13], immunity [14], cancers [15], and the brain–gut–immune axis [16]. At the phyla level, the bacteria in the human gut are mainly composed of Firmicutes and Bacteroidetes; others are Actinobacteria, Proteobacteria, Spirochetes, Synergistetes, Verrucomicrobiota, and Fusobacteria [17,18,19].

The microbial definition of a healthy human gut is debated because of the variation in what constitutes a ‘healthy microbiome’ [11]. However, the answer is being unravelled with the advancement in high-resolution genomic studies such as metagenomics, metatranscriptomics, and metabolomics [20]. The gut microbiota is influenced in the foetus by the maternal diet, health, and exposures; at infancy by the mode of birth, birth term, and feeding; and in childhood and later life by host genetics, lifestyle, diet, age, environment, and medications [21,22,23]. However, microbial diversity and ecological balance is largely accepted as a measure of a ‘healthy gut’ [24].

High sodium intake is a global health concern, primarily because of its association with hypertension and cardiovascular diseases, and could adversely affect other targeted organs even when there is no increase in blood pressure [25,26,27]. The World Health Organization recommends an intake of less than 2000 mg of sodium/day [27]. However, most people around the world still consume too much sodium, which can cause about 1.7 million deaths each year [28]. Dietary sodium is predominantly consumed from salt. The implications of sodium on the gut microbiome and the potential mechanistic pathways through which it may influence host health are not well understood. Emerging research suggests that sodium can alter the gut microbial composition, potentially affecting the host’s immune response, metabolic function, and even the pathogenesis of diseases [29,30,31].

There are minimal human studies that evidence the influence of sodium on gut microbiota as most studies are largely from non-human guts. A high-salt/sodium diet (HSD) has been implicated in the exacerbation of colitis, characterized by a decrease in *Lactobacillus* and butyrate production [30]. Most microbiome reports on dietary salt or sodium have focused on how HSD negatively affects intestinal immunity, and exacerbates colitis, inflammatory bowel disease, and hypertension [29,31,32]. Wang et al. [33] reported that HSDs increase the abundance of *Lachnospiraceae* and *Ruminococcus* but decreased *Lactobacillus*. A pilot study using Wistar rats showed that the high-salt group had a significant reduction in *Lactobacillus* and *Prevotella* NK3B31, and a significant increase in *Alloprevotella* and *Prevotella 9* [34]. Studies by Kumar et al. [35] reported a reduction in the abundance of both Firmicutes and Bacteroidetes in the gut of high-salt-fed rats compared to low-salt guts. In another animal study, loss of *Lactobacillus* and other beneficial genera in Firmicutes was not observed in the high-salt cohort of wilding mice [36], though it was observed in conventional laboratory mice. In a related study on risk factors of hypertension, HSD led to the reduction in beneficial *Bacteroides* (not *B. fragilis*) [37]. A human study by Ferguson et al. [38] showed that HSD is associated with increases in *Prevotella*. There is a research gap in the field of human studies that investigates the impact of sodium on the gut microbiome. While there is a growing body of research on the role of sodium in systemic health, the specific effects of sodium on the gut microbiome remain inadequately explored. Existing human studies primarily focus on the cardiovascular implications of high sodium intake, neglecting the potential interactions with the gut microbial community. Understanding these interactions is crucial because emerging evidence suggests that dietary components, including sodium, can influence the composition and functionality of the gut microbiome, and influence overall health. Consequently, the aim of this study is to assess the influence of dietary sodium on the gut microbiome in human subjects.

In this study, we used human subjects. First, we collected food diaries and faecal samples and then performed a dietary analysis. Thereafter, samples were grouped into a high-sodium diet and low-sodium diet and afterwards taxonomic analyses from the high-resolution shotgun metagenome to understand the compositional uniqueness in both gut types were performed. Then, we explored the distinct abundance of *Bacteroides* and *Prevotella* within the gut ecosystem and examined their potential as indicators of dietary sodium. We also consider how the ratio of these two genera can reflect compositional changes in the gut microbiota. Finally, we use predictive functional tools to investigate the likely relationship between the microbiome and metabolic pathways relevant to sodium reabsorption and cholesterol metabolism.

## 2. Results

### 2.1. Dietary Record and Nutrient Evaluation

Each high-salt/sodium diet (HSD) and low-salt/sodium diet (LSD) group has three samples. Dietary and metagenomic results were based on these six samples (HSD—*n* = 3, LSD—*n* = 3). The average sodium intake for the HSD group was 4534 mg/day while that of LSD was 1058 mg/day. Sodium consumption in HSD is four folds higher than LSD, and two folds higher than the recommended maximum sodium intake (Supplementary S7). This allows for the comparison of the influence of dietary salt intake on the gut microbiota. The AOAC fibre intake for LSD was 17.5 ± 1.95 g/day and HSD was 16.8 ± 5.29 g/day (Supplementary S7). The mean age of the group on HSD was 30 ± 2.08 years while those classified as LSD were 44 ± 5.24 years. All the participants were residents in England and were females except a male participant in the LSD group. A detailed nutritional analysis is available in the supplemental information (Supplementary S1).

### 2.2. Taxonomic Analysis of Microbial Communities in High-Salt Gut and Low-Salt Gut

**Figure 1:**
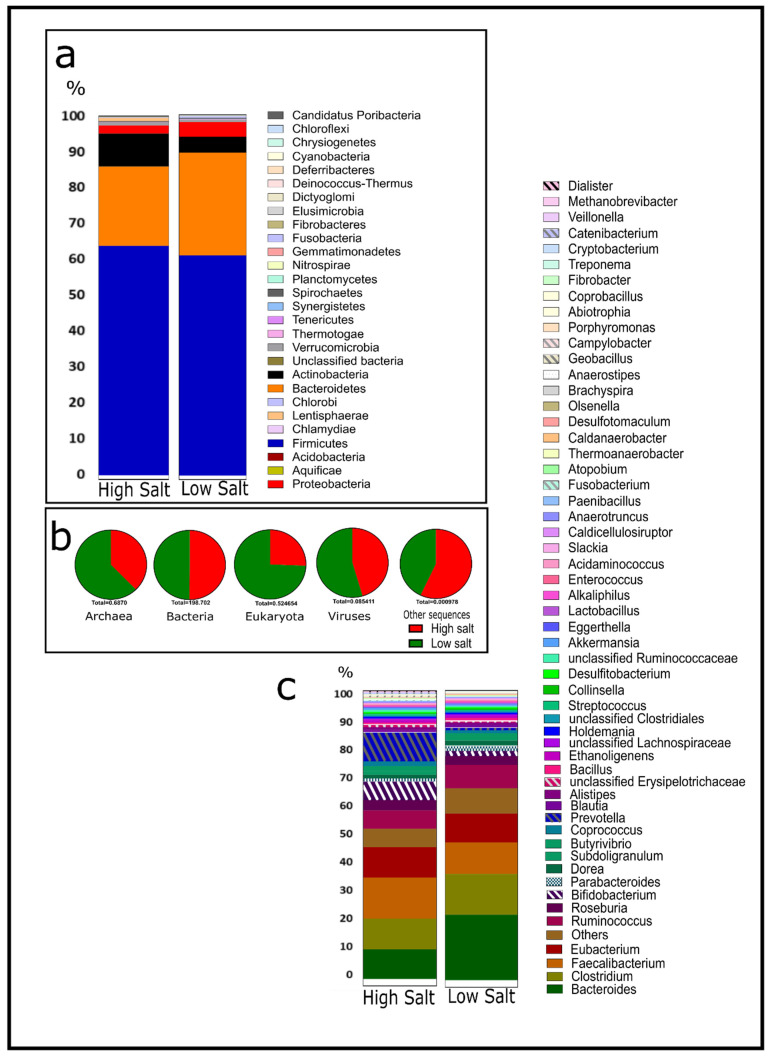
Metagenomic microbial community analysis of the human guts by salt intake classification. (**a**) Relative abundance at phylum level. (**b**) Proportions of the domains. (**c**) Relative abundance of top fifty-five genera. HSD, *n* = 3; LSD, *n* = 3.

In the life-level domain, there is a close proportion in the relative abundance of bacteria irrespective of diet groupings—HSD (99.6 ± 0.03) and LSD (99.2 ± 0.21) (Figure 1b). In both groups, the dominant phylum is Firmicutes (61.8 ± 1.4), followed by Bacteroidetes (24.7 ± 3.2), Actinobacteria (5.9 ± 2.4), Proteobacteria (3 ± 0.9), and Verrucomicrobia (0.7 ± 0.01) (Figure 1a). This is similar to studies by Sánchez et al. [17], Shkoporov and Hill [18], and Zhang et al. [19]. An alpha diversity analysis revealed slightly higher microbial diversity in LSD (Shannon index: 3.23) when compared to HSD (Shannon index: 3.17) (Supplementary S2a). Non-metric Multidimensional Scaling (NMDS) by a Bray–Curtis dissimilarity matrix indicated that HSD samples were more loosely grouped based on relative abundance at the genus level than LSD (Supplementary S2b). Previous studies by Ferguson et al. [38] also indicated like ours no significant difference in the alpha diversity in LSD versus HSD. Their NMDS results also mirrored ours and indicated no significant differences in bacterial clustering between LSD and HSD. This suggests individual variation in microbial communities for all dietary groups, though variation is higher in high-salt guts. The human gut microbiome is a complex and diverse community of microorganisms, and variation may be due to the individual genetic makeup, health status, age, diet, immune response, microbial interactions, environment, and lifestyle [21,22,23]. In both groups, the dominant genera include *Bacteroides*, *Clostridium*, *Faecalibacterium*, *Eubacterium*, *Ruminococcus*, and *Roseburia.* Figure 1c shows the top 55 genera across the groups.

**Figure 2:**
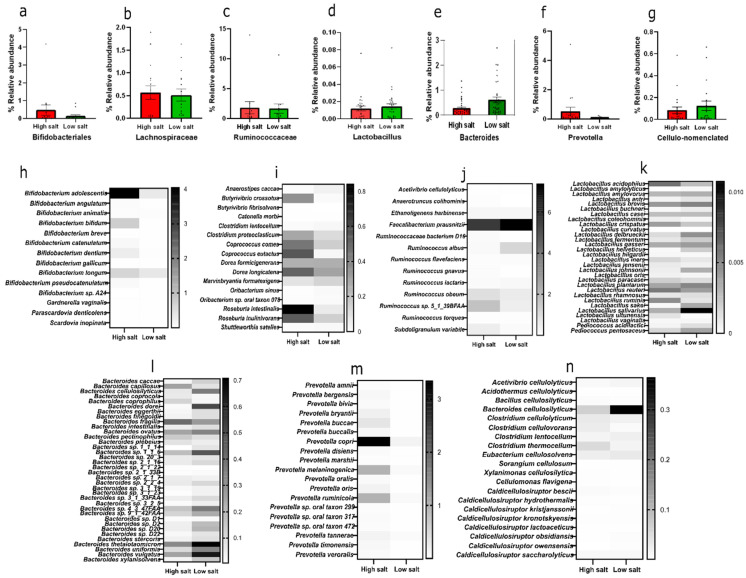
Relative abundance of selected commensal microbes. Top row—relative abundance of (**a**) Bifidobacteriales, (**b**) Lachnospiraceae, (**c**) Ruminococcaceae, (**d**) Lactobacillus, (**e**) *Bacteroides*, (**f**) *Prevotella*, (**g**) cellulo-related. Middle and bottom row—heatmaps of members (**h**) Bifidobacteriales species, (**i**) Lachnospiraceae species, (**j**) Ruminococcaceae species, (**k**) *Lactobacillus* species, (**l**) *Bacteroides* species, (**m**) *Prevotella* species, (**n**) cellulo-related species. Calculated as relative abundance with standard error of means within the bacteria domain. HSD, *n* = 3; LSD, *n* = 3.

By class, HSD has a higher abundance of Bifidobacteriales than LSD (Figure 2a) and this is chiefly *Bifidobacterium adolescentis* (Figure 2h). Bacteria that are cellulose-related in nomenclature constitute about 2.8% in the low-salt gut compared to 1.7% in the high-salt gut (Figure 2g). To the best of our knowledge, this is the first report about the abundance of cellulose-related bacteria in the gut in relation to dietary salt. The mean AOAC fibre consumed by an HSD recipient was 16.8 g/100 g and LSD was 17.5 g/100 g (Supplementary S7). Despite a similar overall fibre consumption pattern in both groups, these cellulose-degrading bacteria are more abundant in the low-salt gut and chiefly dominated by *Bacteroides cellulosilyticus* (Figure 2n). Other comparative taxonomy includes Lachnospiraceae, dominated by *Roseburia intestinalis* (Figure 2b,i), and Ruminococcaceae, dominated by *Faecalibacterium prausnitzii* (Figure 2c,j), *Lactobacillus* (Figure 2d,k), *Bacteroides* (Figure 2e,l), and *Prevotella* (Figure 2f,m).

### 2.3. Significant Microbial Shift

**Figure 3:**
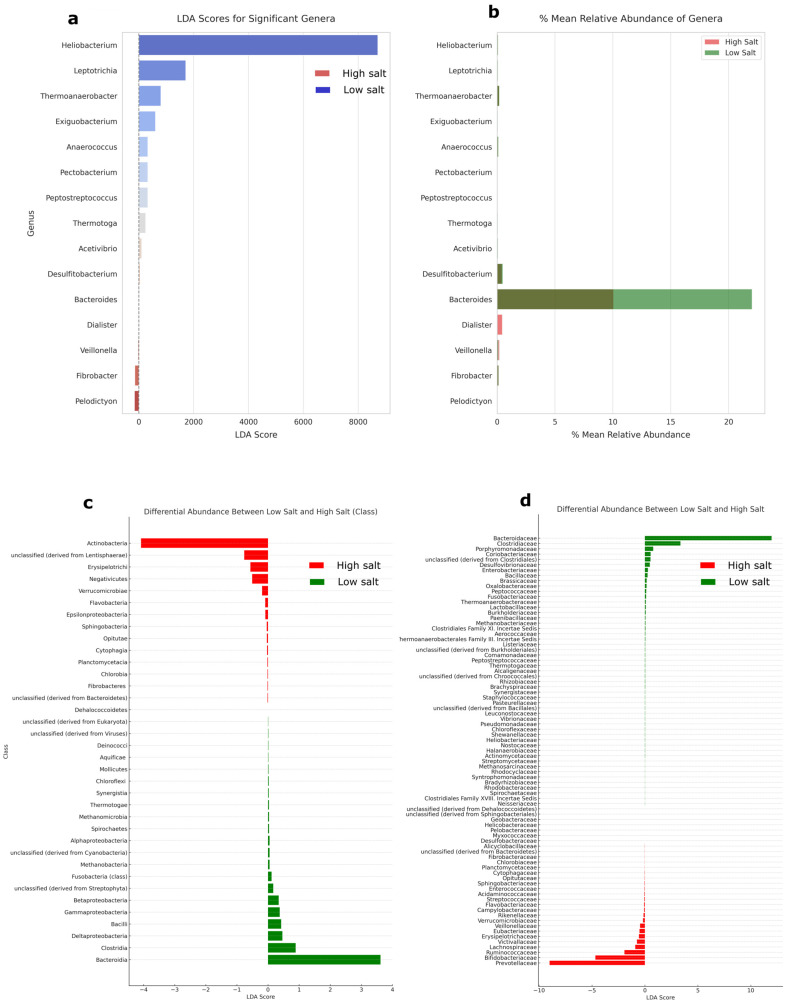
Linear discriminant analysis. (**a**) Kruskal–Wallis test was performed to identify genera with statistically significant differences in abundance between the high- and low-salt groups (*p* = 0.05). (**b**) Relative abundance of significantly differential genera. (**c**) Mean differential abundance between HSD and LSD at class level. The total class number was 126, and classes with less than 0.05% abundance were filtered out before calculation of differential abundance with significance across the two groups. (**d**) Mean differential abundance between HSD and LSD at family level. The total family classification was 416, and families with less than 0.05% abundance were filtered out before calculation of differential abundance with significance across the two groups. Further results on genus-level *p* values for Shapiro–Wilk test and Krustal–Wallis test are available in Supplementary S5. HSD, *n* = 3; LSD, *n* = 3.

A linear discriminant analysis revealed that there are significant differences between HSD and LSD at the genus level. *Heliobacterium* had the highest LDA score of 7200 and positively leaned towards LSD (Figure 3a). Other significant microbes for LSD include *Leptotrichia*, *Thermoanaerobacter*, *Exiguobacterium*, *Anaerococcus*. Despite a low LDA score for *Bacteroides*, the genus had the highest relative abundance among the differential genera (10% for HSD and 23% for LSD) (Figure 3b). Because of their high abundance and differential significance in HSD and LSD, investigating their ratios or microbial shift may be important in understanding diet–microbiome functions and interactions.

**Figure 4:**
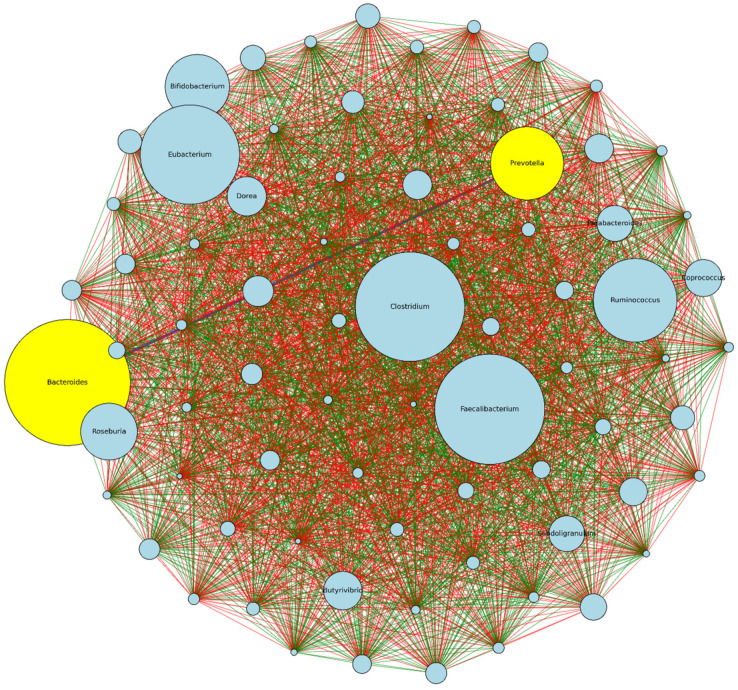
Correlation and network analysis of genera. Each node (circle) represents a genus, and the size of the node corresponds to the average abundance of the genus across both dietary classifications. The lines (edges) connecting the nodes represent the strength and direction of the correlation between the connected genera. Green edges (-) indicate a positive correlation. Red edges (-) signify a negative correlation. HSD, *n* = 3; LSD, *n* = 3.

The visualization in Figure 4 represents a network analysis based on the pairwise Spearman correlations between genera across the HSD and LSD conditions. The more abundant genera are *Bacteroides*, *Faecalibacterium*, *Clostridium*, *Eubacterium*, *Ruminococcus*, *Prevotella*, *Bifidobacterium*, and *Dorea*. Others are *Coprococcus*, *Parabacteroides*, *Subdoligranum*, *Butyrivibrio*. Hence, microbial shifts in any of these abundant genera could be investigated in regards to the dietary intake and functional microbiome. From the analysis, *Bacteroides* and *Prevotella* have a negative correlative relationship, implying that as the abundance of one genus increases in one dietary type (e.g., *Bacteroides* in LSD), the abundance of the other genus tends to decrease (e.g., *Prevotella*). Bacteroidia (class) and Bacteroidaceae (family) have the highest mean differential abundance of 3.6% and 11.9%, respectively (Figure 3c,d).

**Figure 5:**
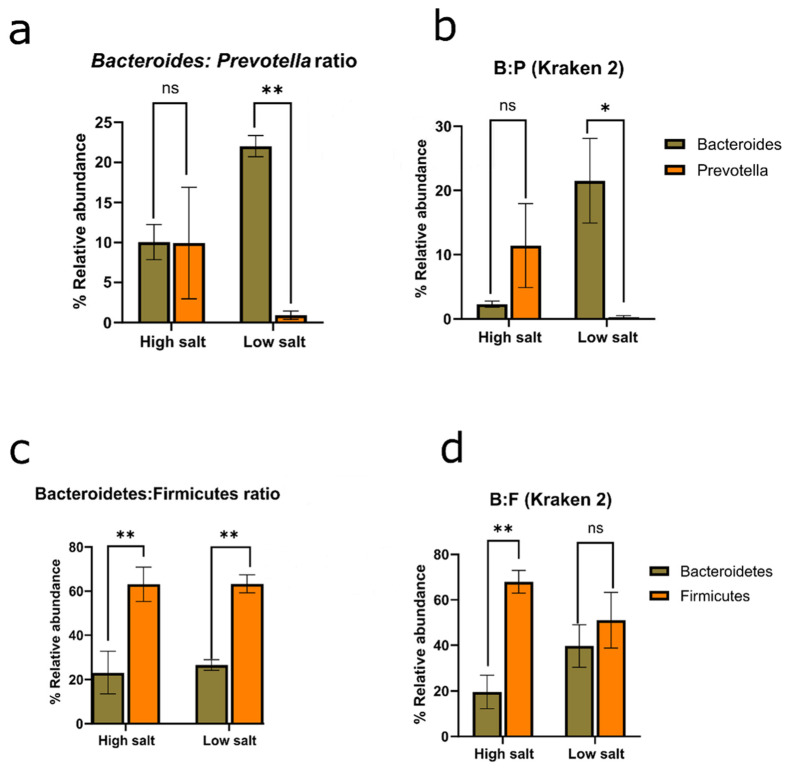
Microbial ratios. (**a**) *Bacteroides*/*Prevotella* ratio based on MG-RAST. Interaction *p* value = 0.0114. (**b**) *Bacteroides*/*Prevotella* ratio based on Kraken 2 taxonomic reports. Interaction *p* value = 0.0224. (**c**) Bacteroidetes/Firmicutes ratio based on MG-RAST. Interaction *p* value = 0.8143. (**d**) Bacteroidetes/Firmicutes ratio based on Kraken 2 taxonomic reports. Interaction *p* value = 0.0709. ANOVA was corrected for multiple comparisons with Šídák’s multiple comparisons test. *p* values were interpreted: >0.1234 (not significant or ns), <0.0332 (*), <0.0021 (**). HSD, *n* = 3; LSD, *n* = 3.

Microbiome studies currently use a shift in the Bacteroidetes/Firmicute ratio as a measure of dissimilar conditions (e.g., healthy vs. disease states) [39,40,41]. However, Figure 5c indicates that there is no clear difference between high salt consumption and low salt consumption based on phyla ratios. Both HSD and LSD show similar 2-fold variance for the F/B ratio. Interestingly, there is significant large variation between these two cohorts when a genus-based *Bacteroides*/*Prevotella* (B/P) ratio is applied even when two different taxonomy platforms are compared (Figure 5a,b). This suggests that low dietary salt intake correlates with increased *Bacteroides* and reduced *Prevotella*. In short, analyses of results from both taxonomic tools indicate that there are low chances (1.1–2.2%) of randomly observing interaction in an experiment of this size. Further supportive statistics for genus-based ratios is available in the supplemental information (Supplementary S3).

There is significant ecological interaction and change in the *Bacteroides*/*Prevotella* ratio by dietary sodium intake. Hence, the suggestion here is that the *Bacteroides*/*Prevotella* ratio should be checked alongside the Bacteroidetes/Firmicutes ratio. Since the consumption of high sodium is known to be unhealthy [27,28], this work proposed that the *Bacteroides*/*Prevotella* ratio could be used complimentarily with the Bacteroidetes/Firmicutes ratio in microbiome studies and investigating a ‘healthy gut’. There is a higher *Bacteroides*/*Prevotella* ratio in a low-sodium gut, which is at least 15-fold. However, a validation analysis would require larger sample sizes and studies performed in various physiological states to elucidate if the *Bacteroides*/*Prevotella* ratio will be a more clinically and statistically accurate ratio rather than the phyla-based Bacteroidetes/Firmicutes ratio for a compositional change marker and the determination of health indices [42].

### 2.4. Functional Predictions

**Figure 6:**
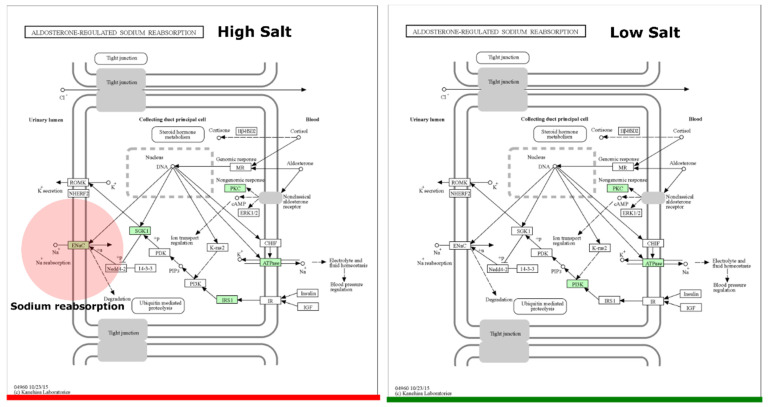
Aldosterone-regulated sodium reabsorption pathway. The ortholog of interest is the epithelial sodium channel (ENaC). Copyright permission obtained for the use of KEGG output figures. HSD, *n* = 3; LSD, *n* = 3.

**Figure 7:**
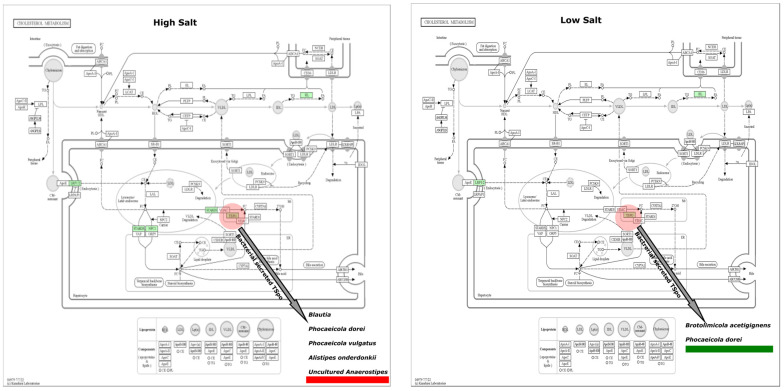
Cholesterol metabolism pathway. The ortholog of interest is the translocator protein (TspO). Copyright permission obtained for the use of KEGG output figures. Reconstruction of all good-quality, complete genomes of *Alistipes onderdonkii* (*n* = 7), *Blautia* (*n* = 31), *Phocaeicola dorei* (*n* = 17), and *Phocaeicola vulgatus* (*n* = 10) available in the BV-BRC/PATRIC also confirms the presence of the TspOs in the genome of organisms above. *Brotolimicola acetigignens* belongs to a new genus with no complete genome [45]. HSD, *n* = 3; LSD, *n* = 3.

The average predicted gene length for both HSD and LSD was 146bp. There was a higher sequence count in LSD (464,813) than HSD (106,752). Similarly, the base pair count was higher in LSD (45,143,166) than HSD (15,645,478) (Supplementary S6). Functional prediction showed that uncharacterised genes were present in the epithelial sodium channel of high-sodium guts but not low-sodium guts (Figure 6). To the best of our knowledge, this is the first microbiome report to find a significant link between high sodium intake and the SCNN1B and SCNN1G gene of the epithelial sodium channel (ENaC). The sequences found in ENaC do not match with any protein in the protein data bank (PDB), Swiss Prot, reference protein (RefSeq), and environmental metagenomic protein (env_nr) (Table 1). The SCNN1G gene (gamma subunit) and SCNN1B (beta subunit) are two of three genes that create the ENaC channel, with the last being SCNN1A (alpha subunit) [43,44]. When compared to the canonical sequence P30536-1 in UniProt using Clustal 2.1 alignment, the *Brotolimicola acetigignens* TspO isoform from our study has been found to have the higher similarity (35.76%) than one of the three computational isoforms B1AH88-1 (31.63%). *Alistipes onderdonkii*, Blautia, *Phocaeicola dorei*, *Phocaeicola vulgatus*, *Brotolimicola acetigignens* were linked to TspO in the cholesterol metabolic pathway (Figure 7) and their corresponding computational structures (Supplementary S8).

## 3. Discussion

Based on previous extensive assessment of microbiota studies, other studies have shown that it is difficult to associate the Firmicutes/Bacteroidetes ratio with a determined health status [40,46]. A clear suggestion was to explore compositional changes at the family, genus, or species level, which might be more relevant than the phyla-based Firmicutes/Bacteroidetes ratio [40,47].

Hence, our study suggests the use of *Bacteroides*/*Prevotella* as a compositional change marker. It is well established that *Bacteroides* and *Prevotella* are the two most abundant genera in the Bacteroidetes irrespective of heathy or disease states in breast cancer [48], blastocystis infection [49], HIV infection [50], urolithin metabotype cardiovascular risk [51], and diabetes [52].

The dominance of these genera in the phylum and indeed the gut microbiome makes them ideal to explore for microbial changes in various physiological states. For instance, studies by An and colleagues [48] reported that the F/B ratio was three times lower in patients with breast cancer than in healthy controls. Indeed, there was about a threefold increase in Bacteroidetes in the disease cohort compared to the heathy cohort; however, their work showed that there were only minor changes in the Firmicutes. In the healthy cohort, mean relative abundance was Firmicutes at 33% and Bacteroidetes at 7% compared to cancer (Firmicutes at 30%, Bacteroidetes at 16%). From the results in their work, a distinct microbial shift may be better reported if the genus-based ratio is applied—healthy (*Bacteroides* at 33%, *Prevotella* at 30%) and cancer (*Bacteroides* at 72%, *Prevotella* at 10%). Nonetheless, not all studies on microbial ratios present genus-level data. For instance, studies by Koliada et al. [39] reported that obese adults have a significantly higher level of Firmicutes and lower level of Bacteroidetes compared to healthy-weight and lean adults. Further inference may have been obtained if results on genera abundance were presented.

Previous works by Gabrielli et al. [49] support our position for the use of the genus-based ratio. In their work, the microbial relative abundance of the blastocystis free group of patients was *Bacteroides* at 32% and *Prevotella* at 2% compared to blastocystis carriers (*Bacteroides* at 1%, *Prevotella* at 11%). *Bacteroides* and *Prevotella* were found to be major bacteria clusters in their investigation on intestinal disorders. Even at the family level, their work showed an inverse ratio of Bacteroidaceae and Prevotellaceae in the control vs. infected. In a viral infection study, the faecal microbiota of untreated individuals with chronic HIV infection exhibited a significantly higher abundance of *Prevotella* compared to HIV-negative individuals (control). Inversely, HIV-negative individuals had increased *Bacteroides* compared to those infected [50]. In a diabetes study, the LDA analysis shows that *Bacteroides* (differential for control) and *Prevotella* (differential for type 2 diabetes) were the two most significantly differential genera [52]. Additionally, these two genera were found to be significantly pronounced in association studies of host factors and microbiome diversity within different compositional clusters [53]. *Prevotella*- and *Bacteroides*-rich compositions were found to be relatively non-overlapping.

Other studies on enterotype detection using cross-national clusters showed that *Bacteroides* and *Prevotella* are the two top genera that drive variation in the human gut [54]. Multispecies research across cows, dogs, deer, geese, humans, pigs, horses, chickens, and seagulls also indicated the usefulness of the *Bacteroides*/*Prevotella* ratio [55]. In their studies, a cluster analysis of *Bacteroides*–*Prevotella* community profiles indicates that *Bacteroides*–*Prevotella* populations from samples of the same host species are much closer to each other than to samples from different source species. Aside from the 16s rRNA bacterial community and metagenome sequencing, which covers genus-level composition, investigation using qPCR and RT-PCR may benefit from genomic targeting of *Bacteroides* and *Prevotella* for elucidating who is there in various physiological environments.

To explore what they (microbes) can do, we applied functional tools with a focus on the epithelial sodium channel (ENaC) in the sodium reabsorption pathway and translocator proteins (TspOs) in the cholesterol metabolism pathway because of their relevance to sodium intake. TspOs, also known as tryptophan-rich sensory proteins, are the mitochondrial benzodiazepine receptor (MBR) family of transmembrane protein present in prokaryotes and eukaryotes [1,56,57]. Mammalian TspO is both a biomarker and therapeutic target, and the roles of these proteins are of research interest [58,59,60]. The knowledge on the similarity between bacterial TspO and the mammalian homologues is a growing area. Yeliseev et al. [61] reported 30% sequence similarity and with advancement in genomic tools, improved structural and functional similarity has been inferred [59,62,63]. However, experiments on *Escherichia coli* and *Saccharomyces cerevisiae* showed the absence of TspO, indicating that it is an unimportant protein for biological activity in some organisms [64]. In our metagenome-based work, metabolic pathway reconstruction found TspOs relating to *Phocaeicola dorei*, *Blautia*, *Brotolimicola acetigignens*, *Phocaeicola vulgatus*, *Alistipes onderdonkii*, and uncultured *Anaerostipes*. Most of these were from guts fed with high-sodium food (72%) compared to low-sodium guts (28%). However, the experimental validation of the involvement of these proteins within the metabolic pathway remains to be elucidated and is proposed as a direction for subsequent research endeavours. This will help answer the question of what the microbes and their bioproducts are actually doing.

Three-decade studies on TspO proteins show that their expression and functions relate to stress-induced changes including exogenous stress such as salt [63,65]. Here, the presence of the TspO in both high-sodium and low-sodium guts may suggest a relationship that is not limited to high salt stress alone. It is beyond the remit of this study to determine if the bacterial TspO displayed function as a sensor (e.g., for high salt or sodium), or as a translocator and transporter (e.g., for cholesterol). Despite the current understanding on the TspO relationship with stress-induced situations, it is unclear how TspO itself functions [10,63].

Meanwhile, the presence of sodium reabsorption genes in the ENaC of HSD (but not LSD) may suggest that there might be a response or adaptation mechanism as a result of high sodium in the gut. ENaC is crucial for sodium absorption in various tissues, including the kidneys and gastrointestinal tract [66,67]. An increased presence or activity of SCNN1B and SCNN1G could potentially reflect the body’s effort to maintain sodium homeostasis in the face of high dietary sodium [68,69]. The lack of these ENaC genes in LSD (but not HSD) could be part of a compensatory mechanism that is less active or unnecessary when the sodium intake is lower [70]. Bacterial proteases such as those from *Pseudomonas aeruginosa* and *Serratia marcescens* are capable of activating the ENaC signalling pathway [71,72,73,74]. However, the precise mechanism of ENaC modulation by proteases has not been fully elucidated as the responsible protease(s) for the proteolytic activation of ENaC is yet to be identified in vivo [75]. Notwithstanding, reconstructed metabolic profiles in this study indicate that high sodium intake activates ENaC at the apical membrane via SCNN1B and SCNN1G genes. Previous studies have shown that HSDs increase ENaC activity and sodium absorption, contributing to hypertension via enhanced sodium entry in the kidneys and immune system [13,76,77,78,79,80,81].

There is still limited knowledge of the role of bacterial TspO in different physiological conditions—when, how, and with whom do they function as a sensor, a translocator, or a transporter. Moreover, on the microbial compositional changes observed here, they are not an indication of any disease predisposition or causality. Rather, they are observations on the relevance of genus-based analyses. The genus-based *Bacteroides*/*Prevotella* ratio may be beneficial for microbiome studies. Future microbiome studies will require correlation and regression analyses of the differentially significant genera alongside various indices within a structured causal methodology such as longitudinal studies, randomised control trials, Mendelian randomization, and structural equation modelling.

## 4. Methods

### 4.1. Study Participants

All participants gave written informed consent before starting this study. Stool samples for this study were collected between January 2018 and December 2018. Ethical approval was granted by the School of Applied Sciences Research Integrity and Ethics Committee, University of Huddersfield (SAS-REIC-17-2711-1). Faecal samples were collected from individuals who responded to the research adverts, which were placed on notice boards within the university, SU shops, and specialised food group. Individuals who were in any of these categories as under 18 years, self-reporting as sick, pregnant, not comfortable with collecting their faeces, or using antibiotics within the past three months before the commencement of this study were excluded from this study. An additional inclusion criterion is that participants should demographically be residents in England. The included participants were initially classified as a vegetarian/vegan/raw cohort and Western/omnivore cohort. Participants could withdraw from this study any time.

### 4.2. Food Diary and Dietary Analysis

Participants were given instructions on how to complete a one-week food diary with detailed daily records of all foods and drinks consumed. After the completion of the food diary on day seven, participants hygienically self-collected faecal samples in pre-supplied DNA/RNA shield tubes [82]. Food diaries, collection tubes, and accompanying quick instructions were single-blindly sent to participants and received without names. On arrival, food diaries and samples were further coded with a laboratory ID to eliminate traceability of samples to participants. Dietary information was examined for completeness, and calculations were made for the average daily consumption of total energy, carbohydrates (including sugars), fats (including saturated fats), protein, fibre, vitamins (including vitamin A, thiamin, vitamin C), minerals (including iron, calcium, chloride, and sodium).

Nutrimen, a professional dietary analysis, was used to analyse the food diaries [83]. This platform allows for a quick look at pre-populated nutritional values according to portions. It is based on a data source from Public Health England’s McCance and Widdowson’s The Composition of Foods Integrated Datasets (CoFIDs) and Food Standards Agency food portion sizes [84,85,86]. In rare situations where particular foods are not available in the Nutrimen database, they were manually entered using the nutritional values on product labels, which were checked in grocery stores or websites. After analyses, the samples were appropriately classified into the low-salt diet (LSD) cohort (<2500 mg/day of salt) and high-salt diet (HSD) cohort (>7000 mg/day of salt). The corresponding sodium is LSD at <1500 mg/day and HSD at >2700 mg/day. This reclassification fit into the joint WHO/FAO international recommendations and most country-specific recommendations, which range between 4000 and 5000 mg/day of salt, estimated at <2000 mg/day of sodium [23,27]. Three samples from each cohort were used.

### 4.3. DNA Extraction

Upon arrival, the samples were processed within 24 h. Where brief storage was necessary before processing, the DNA/RNA shield tubes were stored in a fridge. After processing, the remaining samples were immediately destroyed by submerging in a hypochlorite disinfectant and discarded in compliance with Human Tissue Act 2004. Genomic DNA was extracted and purified using Zymo Quick-DNA Faecal/Soil microbe kits (Zymo Research, Irvine, CA, USA), an ultra-high-density BashingBeads™ fracture resistant that omits or reduces the use of organic denaturants and proteinases [87]. Briefly, about 150 mg of the faecal sample was added to the BashingBead™ lysis tube and buffer. Combined chemical and mechanical lysis was then achieved by vortexing at 5 m/s, for 1 min in 5 cycles with a 30 s interval at 25 °C using Bead Blaster 24 (Benchmark Scientific, Sayreville, NJ, USA). The lysed DNA was separated from the cell debris by centrifugation (10,000× *g*). Thereafter, series of filtration lysis, DNA prewashing, gDNA washing, and DNA elution steps were performed. Finally, the eluted DNA was measured using a Qubit^®^ dsDNA HS Assay Kit (Thermo Fisher Scientific, Waltham, MA, USA). The DNA samples were stored at −20 °C for metagenomic sequencing downstream applications.

### 4.4. Sequencing, Quality Checks, and Reads’ Assembling

The extracted DNA passed through quality control (QC) using microfluidics and lab-on-a-chip technology—a Agilent 2100 Bioanalyzer (Agilent Technologies, Santa Clara, CA, USA). Libraries were prepared using a Nextera DNA Flex library preparation kit (Illumina Inc., San Diego, CA, USA). Next, the libraries were normalized, pooled, and paired-end sequenced for system-specific cycles using the Illumina systems (Supplementary S4). Thereafter, a Next-Generation Sequence by shotgun metagenomics was performed on Illumina Hiseq and Novaseq systems (Illumina Inc., San Diego, CA, USA) by Molecular Research LP (Mr DNA, Shallowater, TX, USA). The sequencer produced 2 × 150-bp-paired sequences at 10 million reads per sample. The generated sequencing reads were quality-checked with FASTQC v0.11.8 [88]. After pre-processing stages of trimming, filtering, and removing contaminants, all the samples had high-quality reads. The FASTQ files were assembled into contigs on the command line with St. Petersburg genome assembler toolkits (SPAdes v3.13.1) set with multiple k-mer lengths of 21, 33, 55, 77 [89,90,91]. The assembled contigs were processed for taxonomic classification on MG-RAST [92,93] and Kraken 2 [94], and also used as base files for microbial metabolic pathway analyses on Kyoto Encyclopaedia of Genes and Genomes (KEGG) [43,44].

### 4.5. MG RAST Taxonomic Annotation

The Metagenomics Rapid Annotation using Subsystem Technology (MG-RAST) bioinformatic pipeline offers automated quality control, annotation, comparative analyses, and archiving services [92,93,95]. The assembled FASTA files were uploaded to the server. This was followed by the detection and removal of adapter sequences using a bit-masked k-difference matching algorithm, Skewer [96]. The backend pipelines and processes include screening and removal of *Homo sapiens* host-specific species sequences using DNA-level matching with bowie [97,98]. Thereafter, the sequence files were trimmed by removing low-quality sequences using a modified DynamicTrim [99]. The lowest phred score that was to be counted as a high-quality base was set at a default of 15 and sequences were trimmed to contain at most many sequences below 5. Duplicate Read Inferred Sequencing Error Estimation (DRISEE) was used to assess sequencing quality and noise within the samples [100]. All sequences passed the pre-processing filtration step as checked with FASTQ-MCF. All samples passed the dereplication checks. Next, SortMeRNA, an RNA genecalling tool, was used to search all sequences for potential rRNA genes with a cut-off of 70% identity to the ribosomal sequences from a reduced version of M5RNA [101]. Sequences were clustered with CD-HIT software V4.6.8 using 97% identity for same species clustering [102]. An RNA similarity search was then performed in BLAT, a BLAST-like alignment tool [103]. The parameters include a default e-value of 5, minimum alignment length of 15 bases, minimum abundance adjusted to 2 and searched against representative hits in the RefSeq database (release version 203) [104,105]. Detailed TSV files downloaded were used for the calculation of relative abundance and statistical analyses. Except otherwise stated, all taxonomic reports presented use this pipeline.

### 4.6. Microbial Shift Analyses

A linear discriminant analysis (LDA) was employed to identify bacterial genera that are differentially abundant between high and low sodium intakes. Data were filtered to exclude genera with relative abundances below 0.01%. Initially, the Shapiro–Wilk test was employed to assess the normality of the data distribution. Given the non-normal distribution observed, a non-parametric approach was adopted. The Kruskal–Wallis test was applied to identify genera that exhibited statistically significant differences in abundance between the high- and low-sodium groups (*p* = 0.05). LDA scores were calculated to assess the effect size of differences alongside mean relative abundance providing a comprehensive view on microbial shifts of significance.

### 4.7. Galaxy–Kraken 2 Taxonomic Analyses

For further understanding of the genus-based ratios and pipeline comparison, Kraken 2 (Version 2.1.1) on the Galaxy bioinformatic pipeline was used [106]. The assembled contig FASTA files were inputted, and default parameters include the confident score threshold (0.0), minimum base quality (0), and minimum hit groups adjusted to 2. The database used was Prebuilt Refseq indexes: Standard-16 (Standard with DB capped at 16 GB) (Version: 2022-06-07—Downloaded: 2023-08-17T071759Z). For all samples, Kraken report outputs that assign taxonomic labels to sequencing reads were generated. The reports were viewed and converted into tabular files for the calculation of relative abundance, statistical analyses, and visualization.

### 4.8. Gene Predictions and Metabolic Annotations

To predict genes within the metagenomic sequences derived from stool samples, we employed Prokka v1.13.3, a rapid prokaryotic genome annotation tool [107]. This command-line tool is adept at recognizing coding sequences (CDSs), rRNA, tRNA, and other non-coding RNA molecules, as well as some miscellaneous features, using an amalgamation of various bioinformatics tools. The earlier assembled contigs also served as the input for Prokka. Each sample was processed individually using the default Prokka parameters, which entail a suite of annotation tools such as Prodigal for CDS prediction, Aragorn for tRNA detection, and Barrnap for rRNA identification. Prokka utilized its default database stack, starting with the manually curated Swiss-Prot, followed by RefSeq, and finally relying on computationally predicted annotations from Pfam, TIGRFAMs, and other sources if no match was found in the primary databases.

The output files generated served as input for the KEGG database, a resourceful tool for predictive inference on the functional microbiome [43]. Specifically, the predicted genes were analysed in metagenome-designed GHOSTKOALA for genes that correspond to prokaryotes at the genus level, eukaryotes at the family level, and viruses [44]. Thereafter, pathways were reconstructed for aldosterone-regulated sodium reabsorption (map04960) to understand a potential microbiological link between the amount of sodium intake and sodium reabsorption [108,109]. Likewise, associated cholesterol metabolism (map04979) was reconstructed [110,111]. Finally, the genes found in the biological pathway were pooled together by cohorts and reconstructed again to increase the specificity. The genes and proteins underwent a search against four protein databases (Protein Data Bank, SwissProt, Metagenomic Protein, and RefSeq) for similarity with experimental structures. Finally, those found with significant similarity were reported.

### 4.9. Biostatistics and Visualization

Where relevant, data generated in various genomic pipelines used were pre-processed in Ms Excel, with further statistics and visualization carried out using Graphpad 8, Python v3.12.0 (pandas, scipy, numpy, matplotlib, seaborn), and R v4.3.0 (ggplot2 v3.3.6, phyloseq, tibble, dplyr, ggforce, tidyverse, igraph, gggraph, Hmisc, vegan, BiocManager).

## 5. Conclusions

In conclusion, a high-sodium diet altered the composition of the human gut microbiota with a significant reduction in *Bacteroides* and inverse increase in *Prevotella*. Since it is currently difficult in many microbiome studies to confidently associate the Firmicutes/Bacteroidetes (F/B) ratio with what is considered healthy (e.g., low sodium) or unhealthy (e.g., high sodium), we proposed the use of a genus-based ratio such as the *Bacteroides*/*Prevotella* (B/P) ratio for investigating compositional dynamics of gut microbiota.

## Figures and Tables

**Table 1:** Genes found in the aldosterone-regulated sodium reabsorption and cholesterol pathways. HSD, *n* = 3; LSD, *n* = 3.

Pathway	Kegg Orthology	PDB	Swiss Prot	Metagenomic (env_nr)	RefSeq (refseq_protein)	Closest Neighbour in RefSeq (% Ident)	Gene Length (aa)	Gut Type
Cholesterol metabolism	TspO (K05770)	Yes	Yes	Yes	Yes	*Brotolimicola acetigignens* (100)	161	Low sodium
		Yes	Yes	Yes	Yes	*Phocaeicola dorei* (100)	154	Low sodium
		Yes	Yes	Yes	Yes	*Phocaeicola dorei* (100)	79	High sodium
		Yes	Yes	Yes	Yes	*Blautia* (100)	150	High sodium
		Yes	Yes	Yes	Yes	*Phocaeicola vulgatus* (99.35)	154	High sodium
		Yes	Yes	Yes	Yes	*Alistipes onderdonkii* (93.39)	158	High sodium
		Yes	Yes	Yes	Yes	Uncultured *Anaerostipes* sp. (70.20)	169	High sodium
Aldosterone-regulated sodium reabsorption	ENaC SCNN1B (K04825)	No	No	No	No		196	High sodium
	ENaC SCNN1G (K04827)	No	No	No	No		135	High sodium
		No	No	No	No		63	High sodium

## Data Availability

The original contributions presented in the study are included in the article/Supplementary Material, further inquiries can be directed to the corresponding author.

## Supplementary Materials

The following supporting information can be downloaded at: https://www.mdpi.com/article/10.3390/nu16070942/s1, Supplementary S1: Nutrients analysis. Supplementary S2: Alpha and beta diversity. Supplementary S3: Firmicutes/Bacteroidetes ratio. Supplementary S4: Sequencing librariese. Supplementary S5: Shapiro Wilk Krustal. Supplementary S6: Predicted genes statistics. Supplementary S7: Nutrition statistics. Supplementary S8: Bacterial translocator proteins.
