# Supplementary material for: The Human Gut and Dietary Salt: The Bacteroides/Prevotella Ratio as a Potential Marker of Sodium Intake and Beyond
Source: Nutrients. 2024 Mar 25;16(7):942. doi: 10.3390/nu16070942 (PMC11013828; doi:10.3390/nu16070942)
Supplement: Supplementary file 1 [file nutrients-16-00942-s001.zip › S2 alpha and beta diversity.pdf]

2a

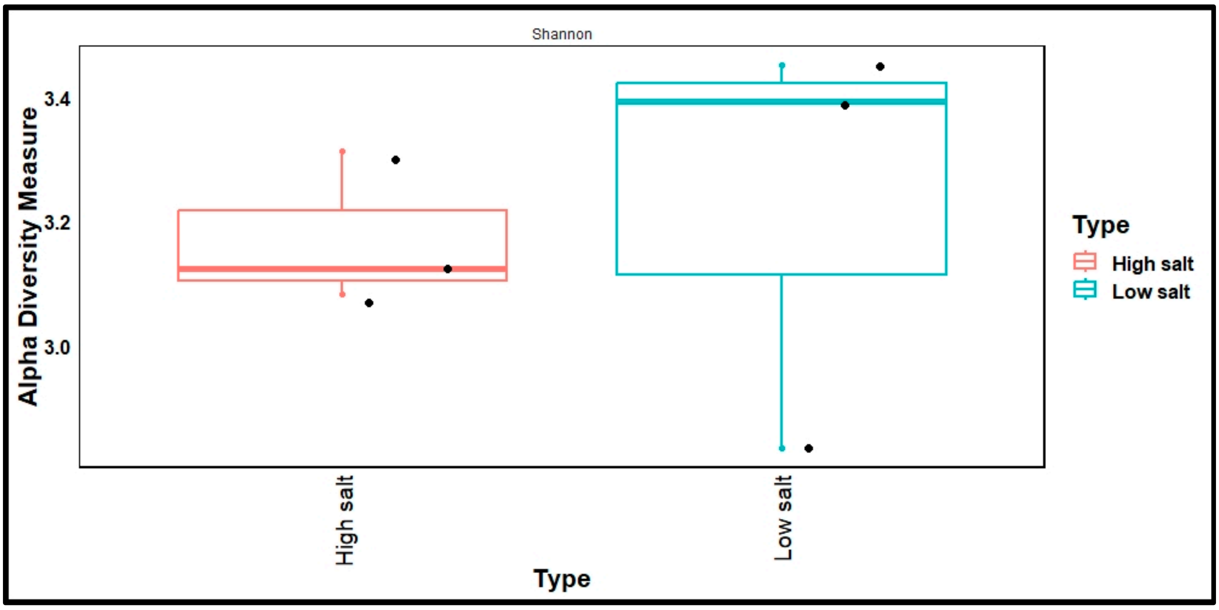

2b

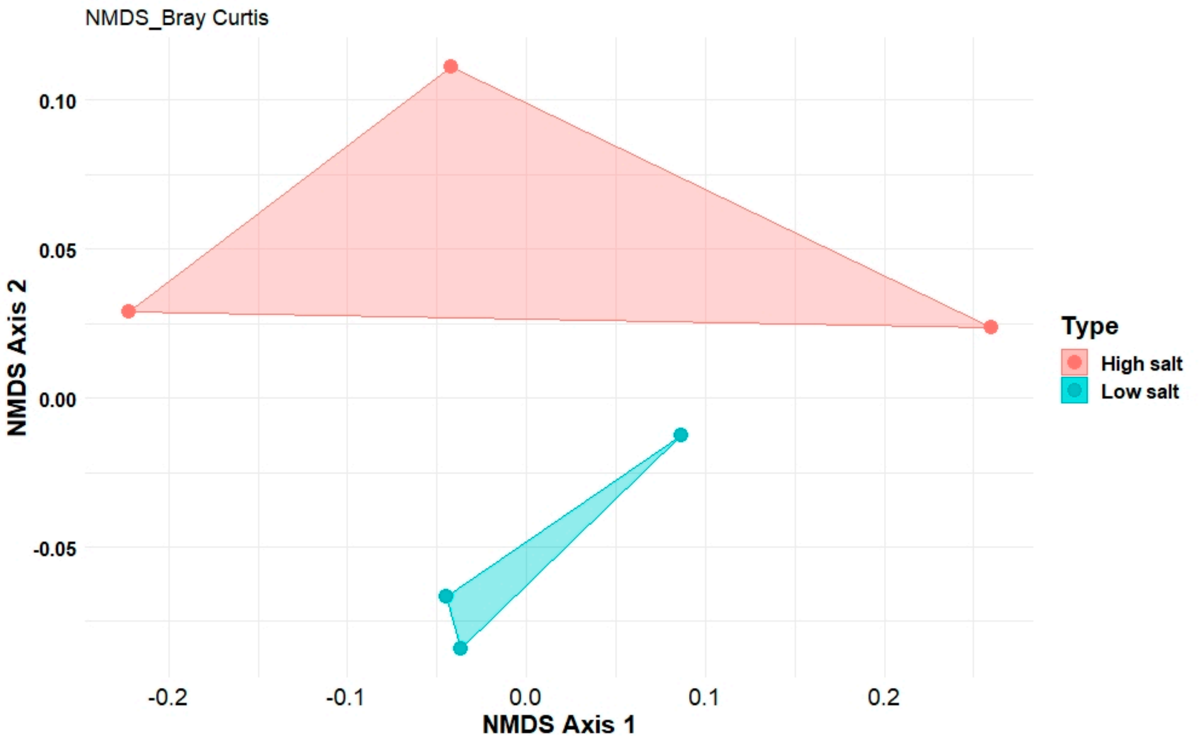

Supplementary 2 (a) Alpha diversity measured by Shannon Index (b) Beta diversity measured by Non-metric Multidimensional Scaling (NMDS)-Bray Curtis. Analyses for the microbial community were based on genus level taxonomy.
