# Supplementary material for: The Human Gut and Dietary Salt: The Bacteroides/Prevotella Ratio as a Potential Marker of Sodium Intake and Beyond
Source: Nutrients. 2024 Mar 25;16(7):942. doi: 10.3390/nu16070942 (PMC11013828; doi:10.3390/nu16070942)
Supplement: Supplementary file 1 [file nutrients-16-00942-s001.zip › S3 Firmicutes Bacteroidetes.pdf]

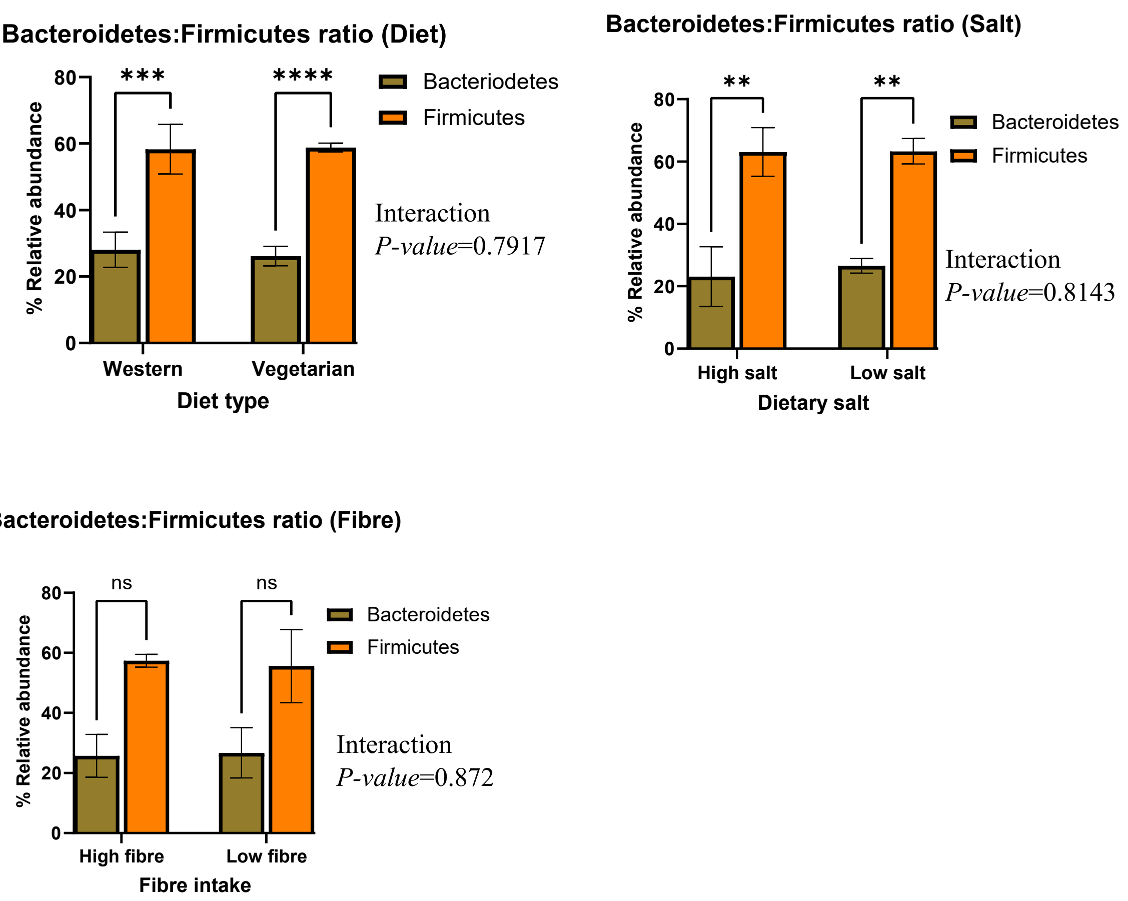

Supplementary 3a: Bacteroidetes/Firmicutes ratios. Western, n=5; Vegetarian, n=5; Low Fibre, n=3; High Fibre, n=3; High Salt, n=3; Low Salt, n=3. Two-way ANOVA corrected for multiple comparisons with Šídák's multiple comparisons test. *P* values interpreted as >0.1234 (ns), <0.0332(*), <0.0021 (**), <0.0002 (***), <0.0001 (****).

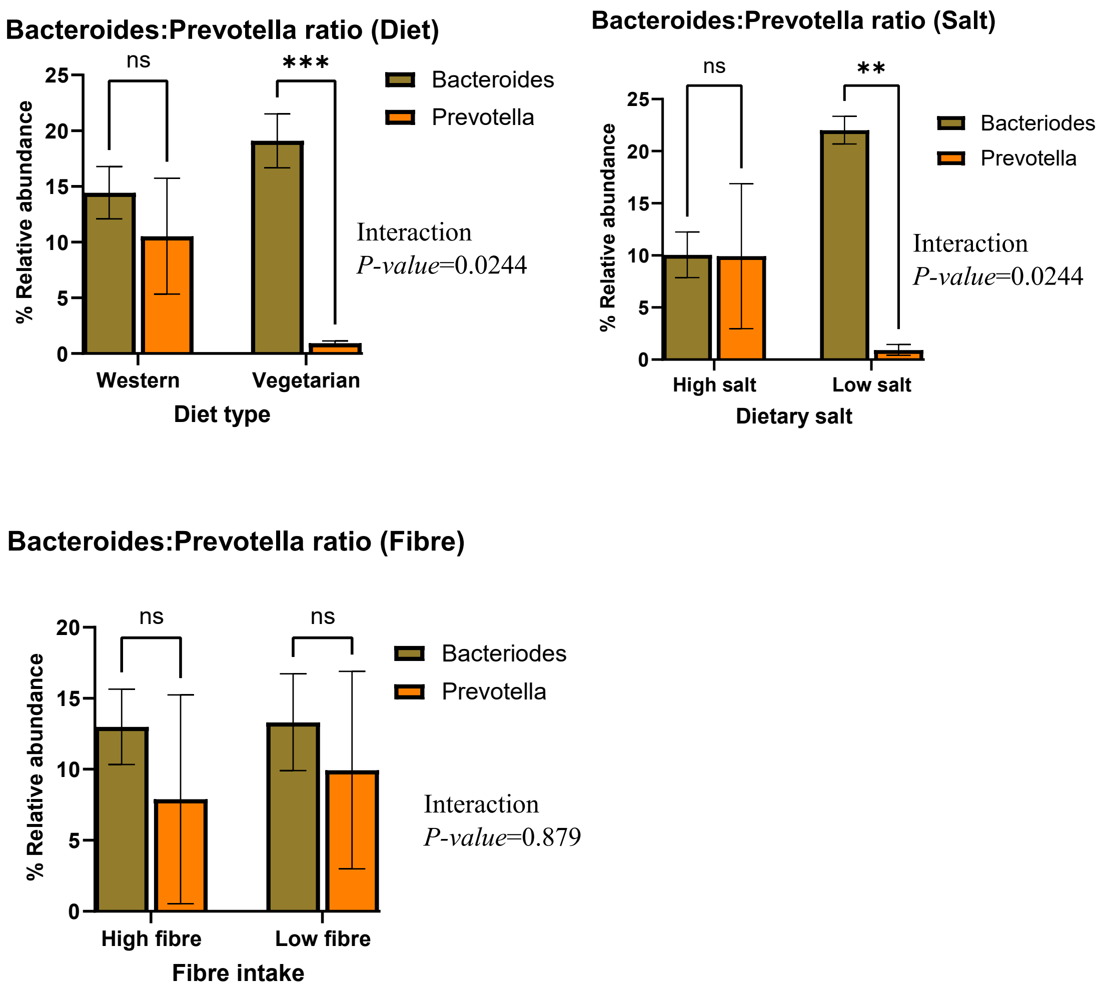

Supplementary 3b: Bacteroides/Prevotella ratio by dietary groups. Western, n=5; Vegetarian, n=5; Low Fibre, n=3; High Fibre, n=3; High Salt, n=3; Low Salt, n=3. Two-way ANOVA corrected for multiple comparisons with Šídák's multiple comparisons test. *p*-values interpreted >0.1234 (not significant or ns), <0.0332(*), <0.0021 (**), <0.0002 (***), <0.0001 (****). Interaction *p*-values were stated.
