# Supplementary material for: The Human Gut and Dietary Salt: The Bacteroides/Prevotella Ratio as a Potential Marker of Sodium Intake and Beyond
Source: Nutrients. 2024 Mar 25;16(7):942. doi: 10.3390/nu16070942 (PMC11013828; doi:10.3390/nu16070942)
Supplement: Supplementary file 1 [file nutrients-16-00942-s001.zip › S4 Sequencing libraries.pdf]

Sample ID	DNA concentration (ng/µL)	Final library DNA concentration (ng/µL)	Average Library size (bp)	Sequencer	Total Cycles	Dilutions of pooled libraries (nM)	No of cycles with adapters
6425	62.80	1.81	437	HiSeq	300	0.01	5
6426	152.0	1.77	388	HiSeq	300	0.01	5
6427	60.0	4.06	450	HiSeq	300	0.01	5
6429	100.0	2.44	386	HiSeq	300	0.01	5
6430	118.0	7.52	516	HiSeq	300	0.01	5
6431	175.00	37.60	595	NovaSeq 6000	300	1	6
3425	86.40	25.80	647	NovaSeq 6000	300	1	6
3426	202.00	34.60	605	NovaSeq 6000	300	1	6
3427	17.50	40.00	585	NovaSeq 6000	300	1	6
3428	135.00	34.60	642	NovaSeq 6000	300	1	6
3431	92.60	12.90	692	NovaSeq 6000	500	0.6	-
Control (water)	116.00*	18.00	690	NovaSeq 6000	500	0.6	-

* Linear amplified DNA. Because of too low DNA concentration for "control" sample, whole genome amplification was carried out by using REPLI-g Midi kit (Qiagen). The linear amplified DNAs were cleaned using DNEasy PowerClean Pro Cleanup Kit (Qiagen) and concentrations were again evaluated.
