# Supplementary material for: The Human Gut and Dietary Salt: The Bacteroides/Prevotella Ratio as a Potential Marker of Sodium Intake and Beyond
Source: Nutrients. 2024 Mar 25;16(7):942. doi: 10.3390/nu16070942 (PMC11013828; doi:10.3390/nu16070942)
Supplement: Supplementary file 1 [file nutrients-16-00942-s001.zip › S6 predicted gene statistics.pdf]

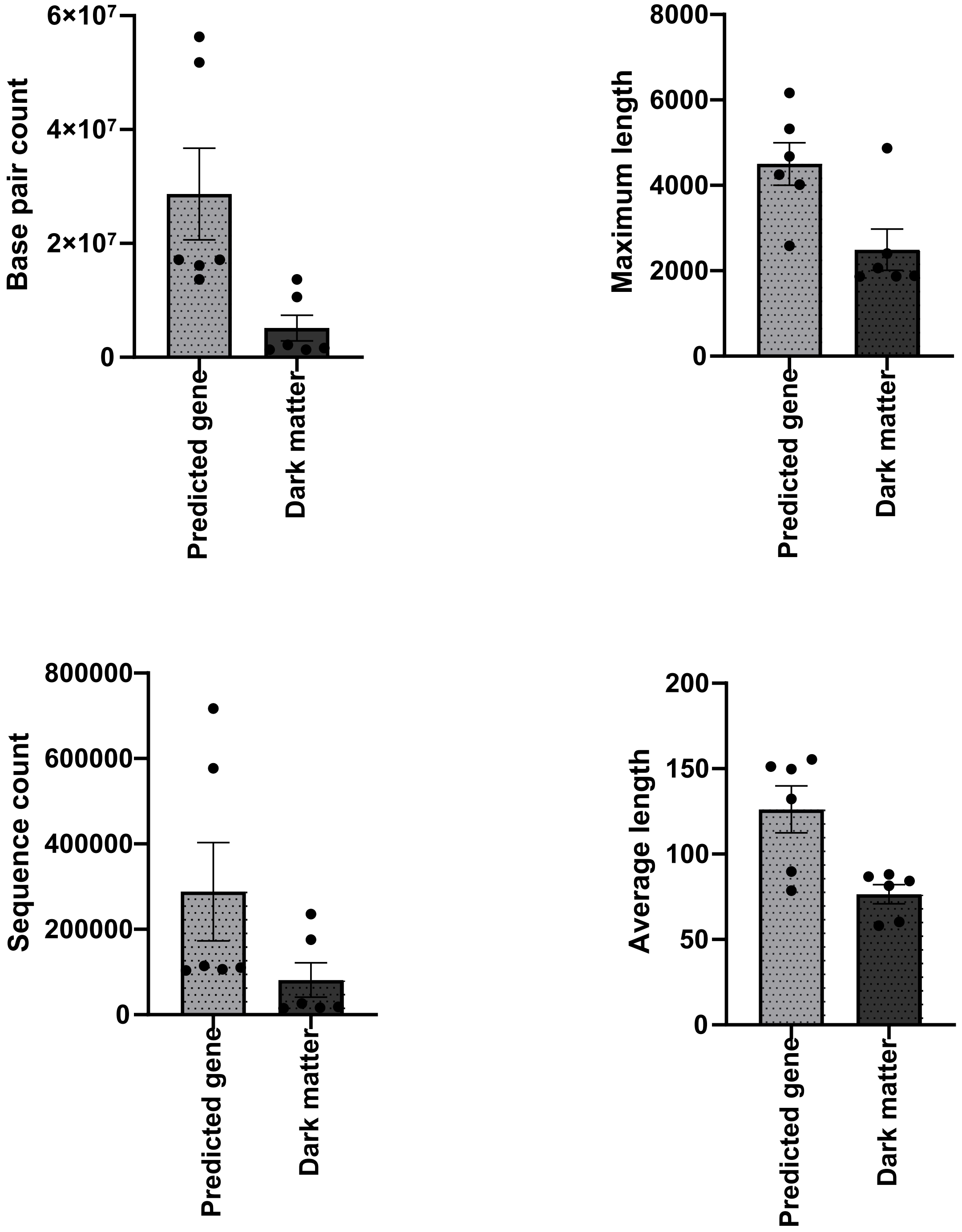


**Figure 2**: **Quality and statistical summary of sequencing and assembling.** **(a)** Base pair count (b) Maximum length (c) Sequence count (d) Average length
