# Supplementary figures and images for: The Human Gut and Dietary Salt: The Bacteroides/Prevotella Ratio as a Potential Marker of Sodium Intake and Beyond
Source: Nutrients. 2024 Mar 25;16(7):942. doi: 10.3390/nu16070942 (PMC11013828; doi:10.3390/nu16070942)

High salt

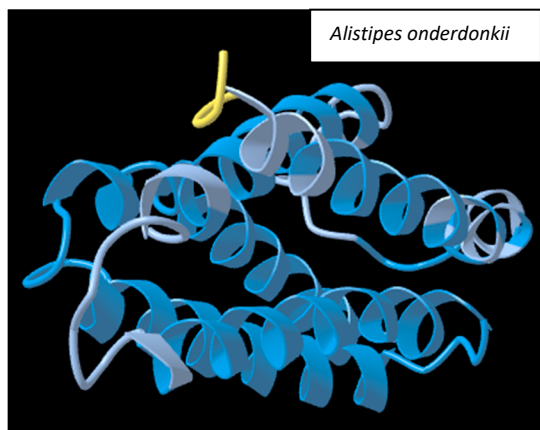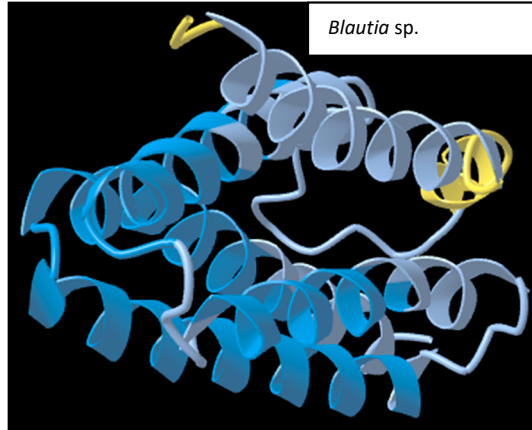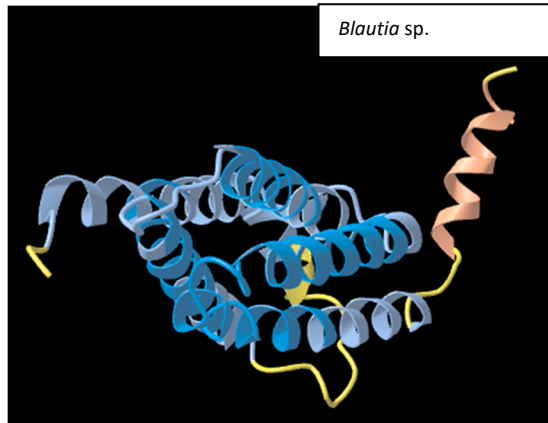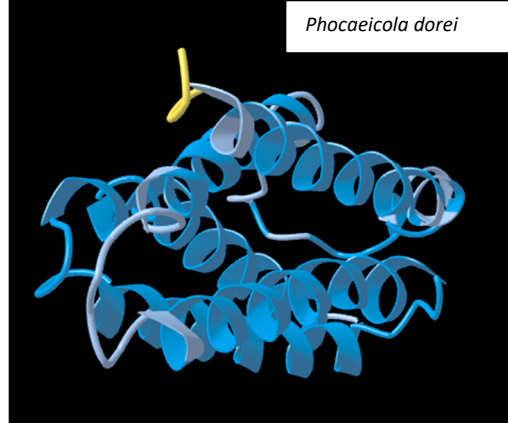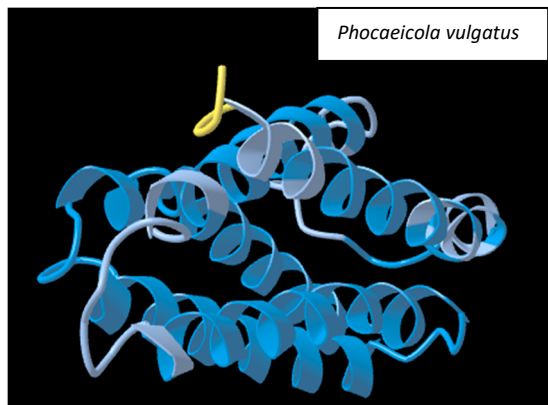

Low salt

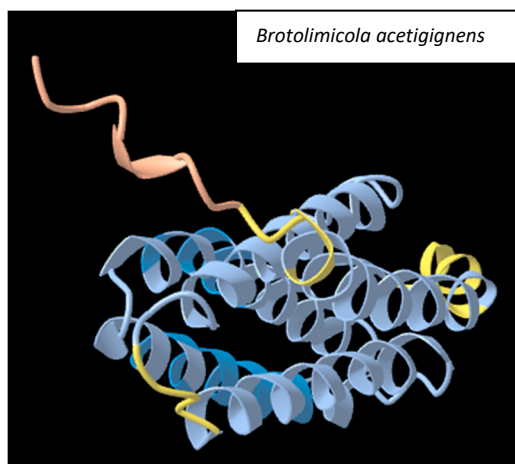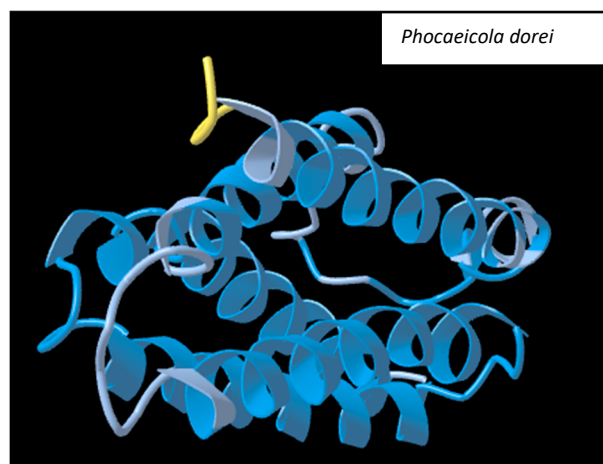

Supplement: Supplementary file 1 [file nutrients-16-00942-s001.zip › S8 TSPOs.pdf]
